# Synchronous bilateral Wilms’ tumor with liver metastasis

**DOI:** 10.1186/s12894-021-00859-8

**Published:** 2021-06-10

**Authors:** Senai Goitom Sereke, Abdirahman Omar Sahal, Vincent Mboizi, Felix Bongomin

**Affiliations:** 1 Department of Radiology and Radiotherapy, School of Medicine, Makerere University College of Health Sciences, Kampala, Uganda; 2 Department of Medicine, School of Medicine, Makerere University College of Health Sciences, Kampala, Uganda; 3 Department of Medical Microbiology and Immunology, Faculty of Medicine, Gulu University, Gulu, Uganda

**Keywords:** Synchronous, Bilateral Wilms’ tumor, Liver metastases, Management challenge

## Abstract

**Background:**

Wilms’ tumor (nephroblastoma) is mostly unilateral; however, bilateral Wilms’ tumors are seen in about 5–8% of patients. This can be synchronous or metachronous. It is uncommon to get liver metastasis from bilateral Wilms’ tumor.

**Case presentation:**

An 8-year-old male Ugandan presented with a history of abdominal swelling and flank pains for 1 year. There was no history of hematuria. Both ultrasound and computed tomography of the abdomen demonstrated multiple solid lesions in both kidneys and a huge solid mass in segments V, VI, VII and VIII of the liver. Histological examination of renal biopsy specimen was favorable for chemotherapeutic regimens. However, following a multidisciplinary tumor board consensus, a nephron-sparing surgery was deemed unsuitable, and he was managed conservatively with chemotherapy (adriamycin and vincristine) with a palliative intent.

**Conclusions:**

Metastatic bilateral Wilms’ tumor has a particularly poor prognosis. There are no clear evidence-based guidelines for the management of this rare presentation. This patient benefited from early palliative care and symptom management.

## Background

Wilms’ tumor, also called nephroblastoma, is the most common malignant renal tumor of childhood, affecting approximately 1 in 10,000 children [1]. It is the most common solid abdominal tumor in children in sub-Saharan Africa [2]. In about 90% of cases diagnosis is made before the age of 7 and most frequently before the age of 5 years [2, 3].

Bilateral Wilms’ tumor accounts for 5–8% of all cases of Wilms’ tumors, which can be synchronous (occurring in both kidneys at the same time) or metachronous (occurring in both kidneys at different time points) [4]. In spite the overall good prognosis of Wilms tumor, the treatment for bilateral Wilms’ tumor remains a clinical challenge [5]. Moreover, it is uncertain whether bilateral nephron-sparing surgery improves prognosis of patients [6].

Wilms tumor with metastasis is a poor prognostic factor for patient’s survival [7]. Occurrence of synchronous Wilms’ tumor with metastasis is not well described in the literature. Herein, we report a case of synchronous Wilms’ tumor with liver metastasis in an 8-year-old Ugandan male.

## Case presentation

An 8-year-old male Ugandan child presented with a 1-year history of a progressive abdominal swelling and flank pains. His mother reported they have been in and out of hospitals with the child due to frequent complains of abdominal discomfort. He was treated with antipyretics and unspecified antibiotics for 6 months prior to their referral to our facility for further investigation and management. There was no history of constipation, hematuria, jaundice, urinary retention, or bleeding diathesis. He was the third born in a family of 5 siblings. There was no history of similar condition in the family. There was no family history of malignancy. His father is a businessman, and the mother is a housewife. His childhood was uneventful.

On clinical examination the child was stable and active (Eastern Cooperative Oncology Group Functional Status 3). His vitals were within the normal limits. Conjunctivae was pink and Chest was essentially normal on physical examination. The abdomen was distended and had mild generalized tenderness. There was a palpable mass, approximately 7 finger breaths in the right upper quadrant, which was firm, non-tender, and not ballotable and did not cross the midline. The mass was dull to percussion and active bowel sounds appreciated. There were no enlarged peripheral lymph nodes.

Complete blood count showed mild anemia with a hemoglobin level of 9.5 g/dl, while the other indices were normal. Renal function test and liver function tests were all normal.

**Fig. 1:**
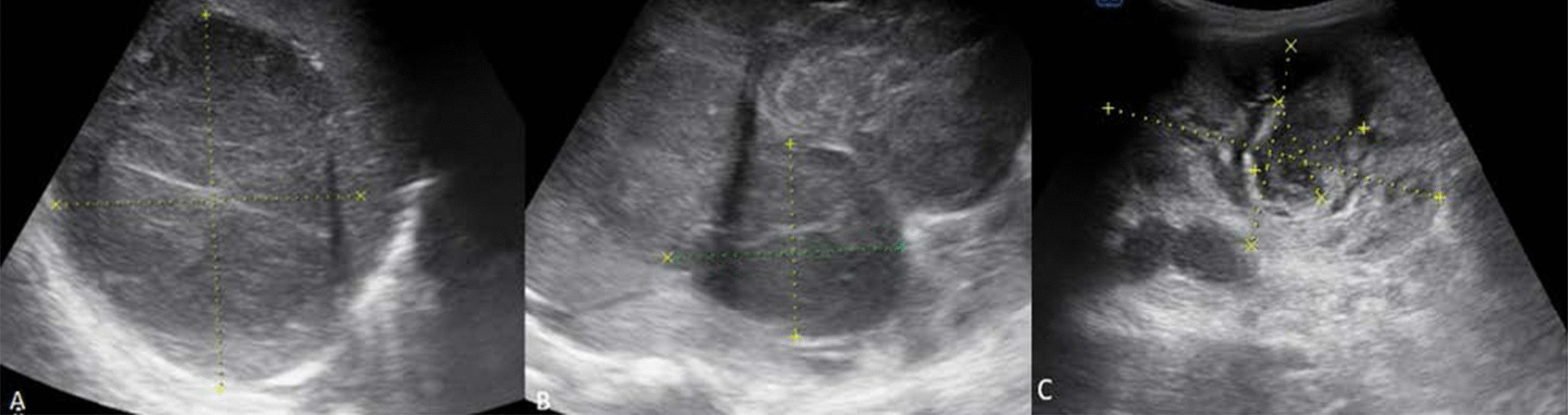
Ultrasound scan of the abdomen. **A** Heterogeneously hypoechoic solid mass in the right lobe of the liver in segments V, VI, VII, VIII; **B** heterogeneous solid mass in the middle to inferior pole of the right kidney; **C** echo complex mass predominantly solid in the left kidney

Ultrasound scan of the abdomen showed a heterogeneous huge mass in the right lobe of the liver, involving segment V, VI, VII and VIII measuring about 11.8 cm by 9.71 cm. The liver was enlarged in size measuring 17.88 cm at mid clavicular line. There was a moderate flow in color Doppler in the mass. Besides there were multiple hypoechoic solid lesions on both kidneys of varying sizes, the largest on the left measured 3.02 cm by 2.55 cm. The inferior venacava showed normal caliber and flow. The origins of the right and left renal veins were visualized but the mid and distal portions were compressed by the masses. There were no enlarged lymph nodes noted in the abdomen (Fig. 1A–C).

**Fig. 2:**
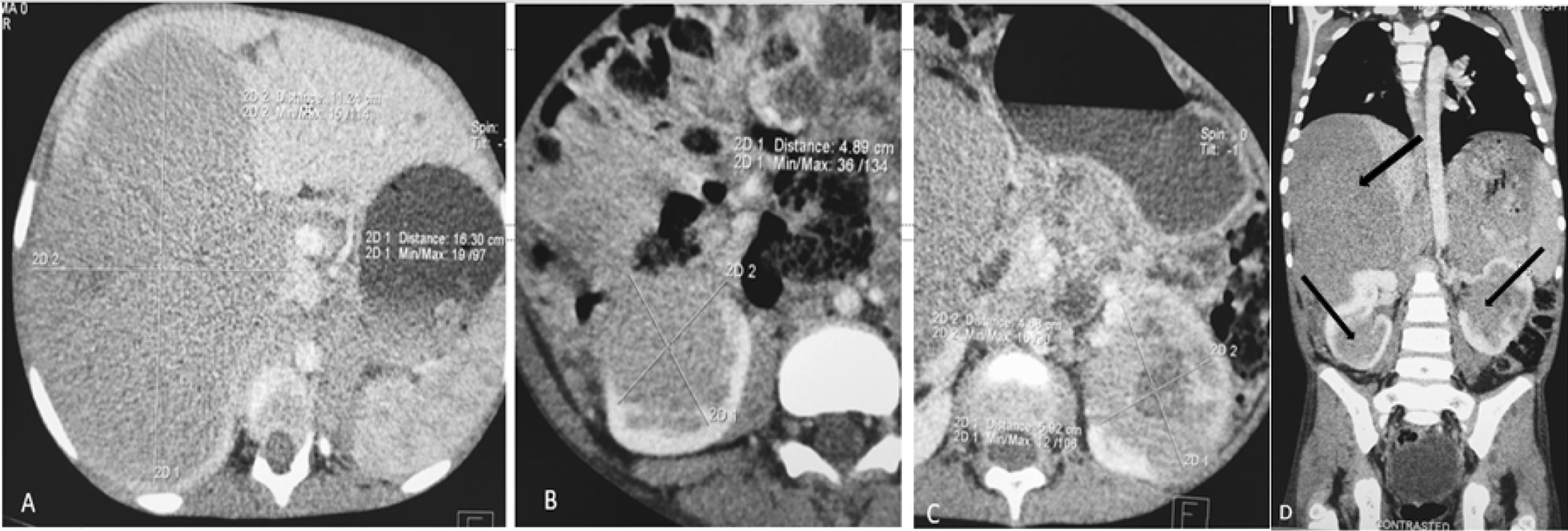
Contrasted CT scan of the abdomen axial (**A**–**C**) and chest, abdomen, pelvis coronal plane (**D**). **A** Homogenously enhancing (35–62 HU) hypo dense solid mass in the segments V, VI, VII and VIII; **B** heterogeneously hypo dense solid mass in the right kidney involving middle to inferior pole; (**C**) hypo dense solid mass with central necrosis involving the whole left kidney; **D** coronal plane of the chest, abdomen, pelvis on soft tissue window demonstrated masses in the liver, right kidney (sparing small portion of the superior pole), left kidney

Contrast enhanced chest-abdomen-pelvis computed tomography (CT) scan showed huge ill-defined, homogeneously enhancing (43–70 HU) hypo-dense solid mass in V, VI, VII and VIII segments of the liver, measuring 16.39 cm by 11.21 cm by 10.2 cm. There was an ill-defined enhancing (30–63 HU) hypo dense solid mass in the right kidney measured 4.89 cm by 4.96 cm by 3.9 cm. There was an ill-defined enhancing (35–62 HU) hypo dense solid mass in the left kidney measured 4.88 cm by 5.92 cm by 4.5 cm with areas of central necrosis. The right renal vein was infiltrated. The right renal artery however was normal. The left renal artery and vein were normal. The ureters and urinary bladder were normal. The portal vein, intrahepatic ducts and common bile ducts were normal. The abdominal aorta and inferior vena cava were normal. There were no enlarged abdominal lymph nodes. There was no ascites. The visualized bones were normal with no focal bone lesion. The radiologic chest anatomy was normal (Fig. 2A–D). Histological examination of renal biopsy specimen was favorable for chemotherapeutic regimens.

In consultation with pediatric oncology tumor board, nephron-sparing surgery was deemed in appropriate given the poor long-term survival even in patients without distant metastasis. Our patient was commenced on combination chemotherapy (adriamycin and vincristine) with a palliative intent. The dose of vincristine was 1.5 mg/m^2^ body surface area administered as intravenous bolus injection and that of Adriamycin was 50 mg/m^2^ BSA infused over 4–6 h were given at 21-day interval. After three cycles of chemotherapy, the abdominal distention had reduced, and the child gained some weight. He remained well until first 6-months from the initiation of his chemotherapy until we unfortunately lost him to follow up.

## Discussion and conclusion

Wilms’ tumor is a common childhood and commonly presents as a unilateral disease. However, bilateral disease is seen in about 5–8% of the cases and can be synchronous or metachronous [4]. Our patient presented with a synchronous bilateral Wilms’ tumor at the time of diagnosis. The patient delayed to receive specialized cancer care and presented with a metastatic disease. In high-income countries, the diagnosis of Wilms’ tumor is usually made at an early stage due to increased awareness and easy access to medical services, hence a better prognosis [8, 9]. Nonetheless, the situation is different in our setting. Uganda is situated in East-Africa and is a low-income country.

Synchronous bilateral Wilms’ tumor presents at a much younger age with a mean age of 2.6 years, compared with 3.3 years in patients with unilateral disease [10]. Our patient presented at the age of 8 years, which is much later than the common age of presentation. The primary distant site for Wilms’ tumor metastases is usually the lung followed by regional lymph nodes and renal vein or inferior vena cava. Hepatic metastases are uncommon [11, 12]. Our patient had hepatic metastases with right renal vein infiltration. However, the lungs were clear and there were no abdominal, hilar or mediastinal nodal involvement.

Size discrepancies of solid tumors were observed when different imaging modalities being used. Studies show that masses measured on ultrasound mostly are larger and some smaller and very rarely the same compared to CT measurements. Ultrasound is a 2D imaging modality and is dependent on the technologist, while CT scan is 3D and least affected by inter/intra-observer reliability [13–15]. In our case, we have found varying results of renal masses as measured by ultrasound and CT scan.

The management of Wilms’ tumor demands multidisciplinary approach. Surgery is considered critical as it is reduces the risk of tumor spread and the need for radiotherapy [1]. Chemotherapy has been incorporated in the management of histologically favorable Wilms’ tumor with good prognosis [1, 16, 17]. In Europe, oncologist prefer to give chemotherapy before surgery (neoadjuvant approach) and in United States it is given post-operatively (adjuvant approach) [18]. Our patient’s renal mass histopathology result showed favorable histology for chemotherapy. However, we were unable to access the histology slides or images.

The management of synchronous bilateral Wilms’ tumor is challenging and has many controversies [19]. There is a high incidence of renal failure occurring in nearly 10% in synchronous and about 20% in metachronous disease. This is way higher than the 1% incidence of renal failure in unilateral Wilms’ tumor. The high incidence of renal failure is mainly due to bilateral nephrectomy [6, 20]. If there is distant metastasis as in the present case, it becomes more complicated. A significant reduction in the size of the tumor can be achieved with the use of pre-operative chemotherapy, making renal salvage surgery feasible [5]. The intent of treatment in this case was palliative rather than cure. Our patient had a huge liver metastasis. He was immediately started on chemotherapy. On his 3rd cycle, the abdominal swelling had reduced. Salvage surgery for the functioning nephrons was supposed to be done after the masses adequately shrank from the original sizes [5, 6]. In some well-established centers, lobectomy is done limited to the affected part of the liver [7, 19]. However, in low-resource settings the postoperative follow up period is compromised due to lack of appropriate resources and economic condition of the patients.

In conclusion, synchronous bilateral Wilms’ tumor is a rare occurrence. Late presentation to a health facility adds up to the existing challenge of lacking treatment guidelines. The current data on synchronous bilateral Wilms’ tumor with distant metastases is scarce. Therefore, management should be patient based until firmly established guidelines are provided.

## Data Availability

The datasets/information used and/or analyzed during this case report is available from the corresponding author on reasonable request.

## Abbreviation

CT: Computed tomography
